# Utilizing hydrophobic sand to construct an air permeable aquiclude to enhance rice yields

**DOI:** 10.1038/s41598-025-87439-0

**Published:** 2025-01-28

**Authors:** Jing Wu, Xiaoyan Ma, Yuming Su, Shengyi Qin, Francesco Pilla

**Affiliations:** 1 State Key Laboratory of Silica Sand Resources Utilization, Beijing, China; 2 School of Architecture, Planning and Environmental Policy, University College Dublin, Dublin, Ireland

**Keywords:** Soil reconstruction, Aquiclude, Hydrophobic sand, Air-Permeable Aquiclude, Rice yield, Water productivity, Environmental sciences, Environmental impact

## Abstract

The Chinese government attaches great importance to the ecological restoration of abandoned open-pit mines, increasing the area of cultivated land, and ensuring food security. Soil reconstruction is a crucial step in ecological restoration of abandoned open-pit mines. This study investigated the utilization of hydrophobic sand to create an Air-Permeable Aquiclude (APAC) under the plant root zones, thereby minimizing water infiltration and enhancing soil aeration. Field plot experiments for 2 years have been conducted, with control groups, Clay Aquitard (CAT), and Plastic Aquiclude (PAC), to evaluate the effects of APAC on rice yield, nitrogen utilization, and water efficiency. The findings revealed that utilizing APAC resulted in a significant rise in rice yield, ranging from 8.09 t/hm² to 9.27 t/hm², which were 7.67–27.16% higher than the control groups. Moreover, the APAC led to a remarkable reduction in irrigation water usage by 37.08%, alongside a substantial boost in Irrigation Water Productivity (IWP) efficiency by 28.64–71.12%. Notably, Nitrogen Partial Factor Productivity (NPFP) exhibited a substantial increase of 7.69–27.06%. These outcomes underscore the APAC’s positive role in water and nutrient conservation and enhanced yields.

**Supplementary Information:**

The online version contains supplementary material available at 10.1038/s41598-025-87439-0.

## Introduction

Mineral resources are an important material basis for human social and economic development, and open-pit mining is the main mining method in China. However, mineral mining inevitably destroys the ecosystem of the mining area, causing severe vegetation destruction and soil erosion, resulting in large-scale soil degradation^1,2^. In 2019, 3.61 million hm^2^ of land was damaged by mining in China^3^, and the number of newly damaged lands continues to increase every year. The Chinese government attaches great importance to the ecological restoration of mines. Since 2016, the China government has deployed 25 ecological protection and restoration pilot projects in 24 provinces, with a total investment of approximately 283 billion CNY. A comprehensive survey and analysis of remote sensing data conducted by China’s Ministry of Natural Resources in 2022 on 2021 data revealed that abandoned open-pit mines in China encompassed 827,400 hm^2^ of mining land, including 26,283.80 hm^2^ of basic farmland. The ecological restoration rate was only 38.67%^4^. Open-pit mining not only disrupts the ecosystem within mining regions but also significantly contributes to the scarcity of arable land resources. Reclaiming abandoned open-pit mine land and converting it into high-standard rice fields has become an important issue that urgently needs to be solved in the ecological restoration and agriculture field in China.

Currently, Research on ecological restoration techniques for abandoned mines has largely focused on soil remediation^5^, phytoremediation^2^, and rewilding^6^, while relatively few studies have explored the potential for converting these areas into rice paddies. This is because the amount of leakage from newly reclaimed rice fields is about 100 times higher than that of rice fields cultivated for more than 100 years^7^. The amount of leakage in rice fields can reach 50–80% of the total water consumption^8,9^, and soil leakage also causes nitrogen fertilizer losses of 38.8–69.99%^10,11^. Meanwhile, it has brought about a series of ecological and environmental problems, such as groundwater pollution and non-point source pollution. Existing research shows that soil reconstruction is the key and foundation for mine land reclamation and ecological restoration. The critical layers of soil, such as topsoil and aquiclude in the soil profile, directly affect the overall function and productivity of the soil system. The aquiclude generally adopts methods such as compacting clay and burying plastic film. The Clay Aquitard (CAT) has poor air permeability, which can easily cause hypoxia in the rhizosphere of crops and affect the normal growth of the root system^12^. Plastic films are mostly high-molecular polymers, which are incompatible with soil properties and difficult to degrade. Currently, they have caused serious pollution to the soil and have an inhibitory effect on crop growth^13^, which can easily lead to a reduction in crop yields. The Plastic Aquiclude (PAC) also isolates the airflow exchange channels of the soil, easily creating an anaerobic environment, and the lack of oxygen in the rhizosphere will seriously affect the growth and development of rice^14,15^. Therefore, constructing an air-permeable aquiclude can not only reduce the inhibitory effect on rice growth but also reduce the amount of soil water leakage, thereby reducing the discharge of fertilizer runoff and polluted water.

Hydrophobic sand has good anti-seepage properties. Research has found that a 2 cm thick layer of hydrophobic sand can withstand a 35 cm high water column^16,17^. Improved air permeability can promote gas exchange at the soil-atmosphere interface and thus increase soil oxygen content^18^. Scholars have used hydrophobic sand to build aquicludes to increase soil moisture and reduce soil erosion^19–21^. In arid regions, the use of hydrophobic sand to construct aquicludes has been shown to enhance both root and above-ground plant growth while reducing irrigation water requirements^20,21^. Moreover, this technique can markedly inhibit the upward migration of heavy metals^22,23^. Hydrophobic sand has also been applied to soil surfaces to reduce water evaporation by 56–78%, increase soil moisture content by 25–45%, and enhance yields of tomatoes, barley, and wheat by 17–73%^24^. Currently, research on using hydrophobic sand to construct aquicludes is primarily focused on cultivating xerophytes, with a lack of research on its application in paddy field reclamation.

This study employs hydrophobic sand to construct an Air-Permeable Aquiclude (APAC), and Clay Aquitard (CAT) and Plastic Aquiclude (PAC) were set side-by-side as control groups. Rice plot planting experiments were conducted in 2021 and 2022 to evaluate the APAC’s effect on rice yield, yield characteristics, and root system characteristics The objective is to reclaim abandoned lands, such as open-pit mines, into rice fields, address food security concerns, and explore innovative technological approaches to enhance water conservation and agricultural productivity.

## Materials and methods

### Study area and materials

**Fig. 1:**
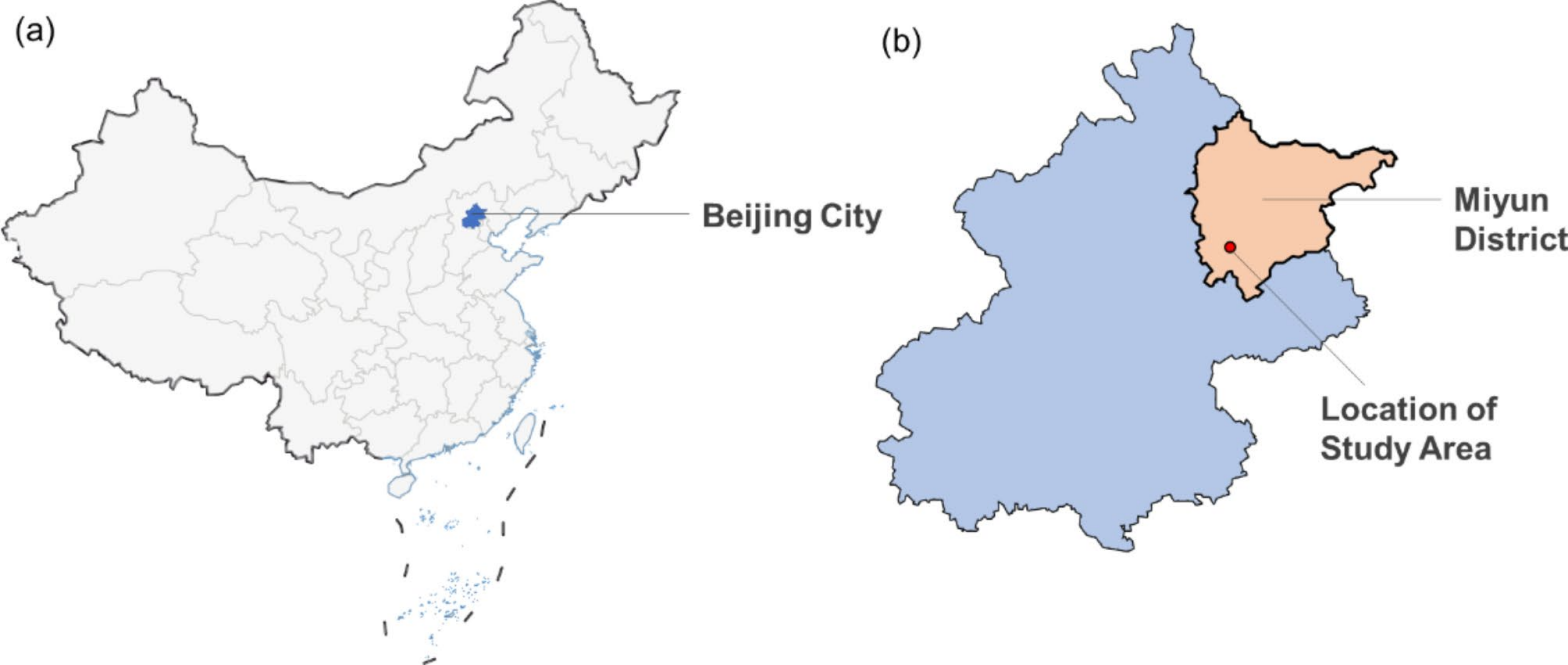
Locations of this study. (a) location of Beijing City in China; (b) location of the study area in Miyun District, Beijing.

**Fig. 2:**
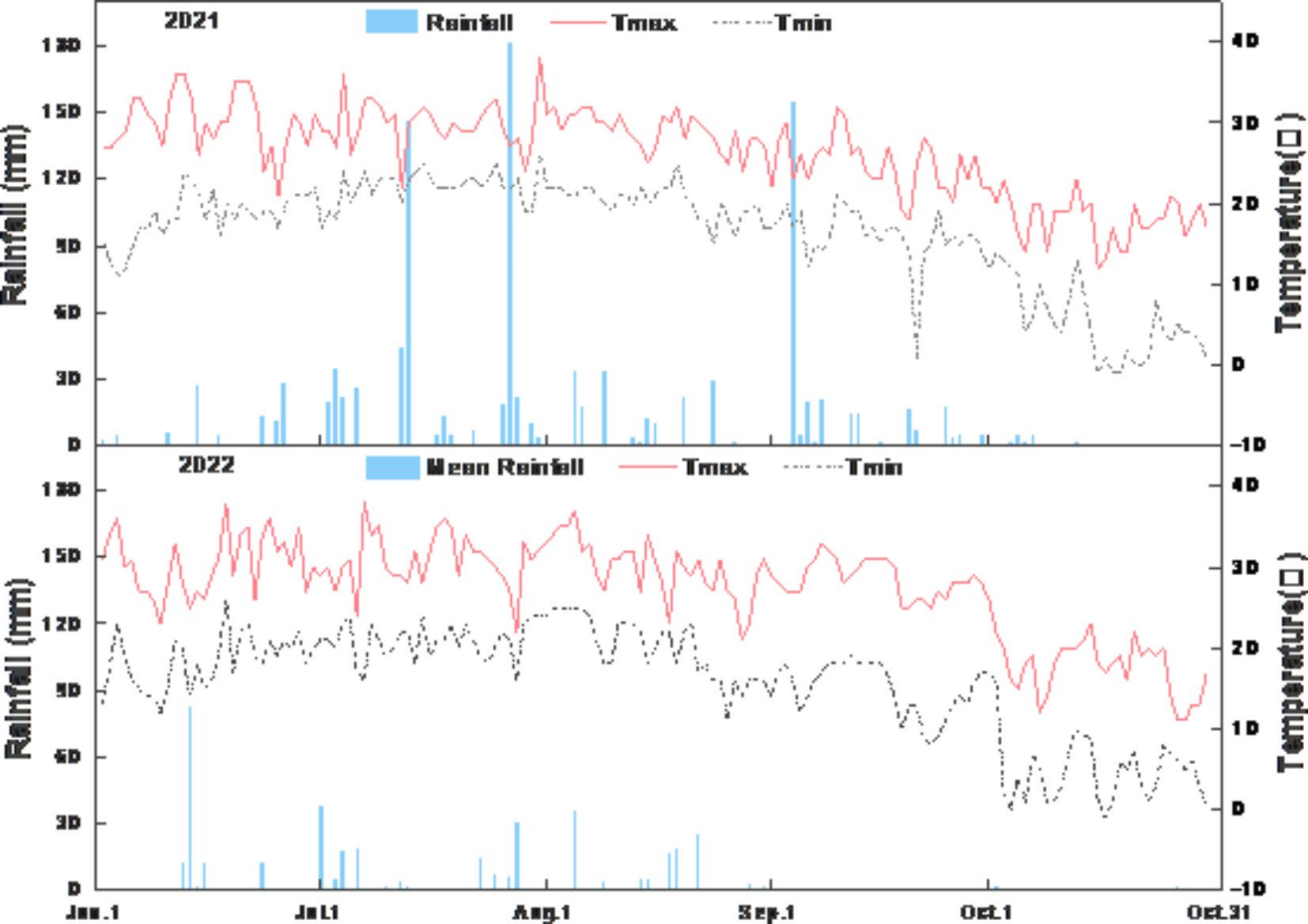
Temperature and precipitation measurements during the rice-growing season in Miyun District, Beijing.

The experiments took place at a pilot farm in Miyun District, Beijing (40°37′N, 116°82′E) (Fig. 1^25^) from June to October in both 2021 and 2022. The temperature and rainfall data during the entire growth period of rice are shown in Fig. 2^26^. The trend of temperature changes after transplanting the seedlings in the two years is similar. Rainfall in 2021 during the growth stage totaled 795 mm, while in 2022, it amounted to 355.5 mm, roughly 44.72% of the previous year’s amount. The in-situ soil is sandy soil with a saturated water holding capacity of 43.27%, pH value of 8.17, organic matter content of 17.4 g/kg, cation exchange capacity of 10.78 cmol/kg, available phosphorus content of 42.8 mg/kg, total nitrogen content of 1.1 g/kg, and available potassium content of 116 mg/kg.

Jingxi rice is a specialty of Shangzhuang Town, Haidian District, Beijing (116°09′35.71″~6°14′06.18″E, 40°04′46.99″~0°09′34.34″N). In 2015, the Ministry of Agriculture of China granted Jingxi rice national agricultural product geographical indication registration protection. In mid-June, Jingxi rice seedlings sourced from nearby farms were planted, transplanted at the site with a spacing of 22.5 cm × 15 cm and 3 to 5 seedlings per hill, and harvested at the end of October.

**Fig. 3:**
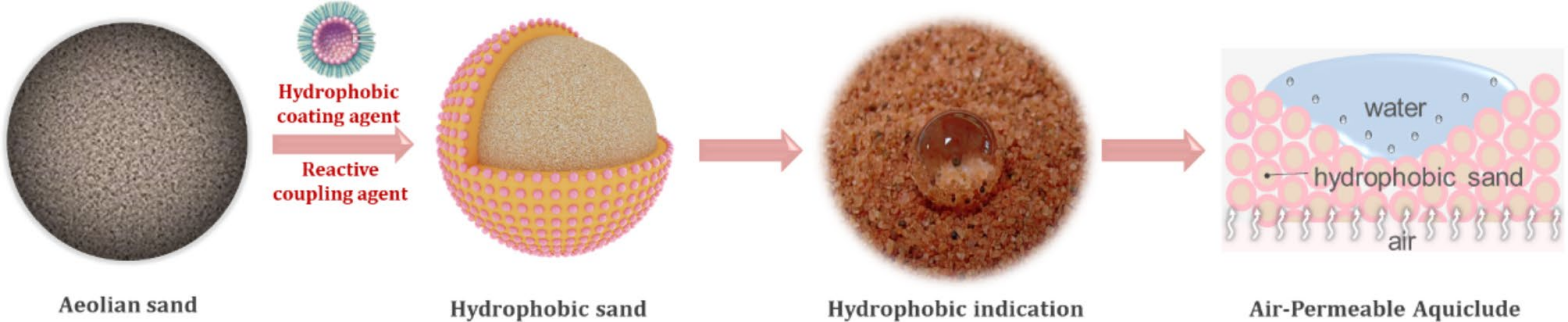
Schematic illustration of surface modification and the construction of the Air-Permeable Aquiclude (APAC).

The hydrophobic sand utilized in this study was obtained from Beijing Rechsand Science & Technology Group Co., Ltd. This sand was employed to construct an Air-Permeable Aquiclude (APAC), wherein the continuous pores formed by the sand particles establish air passages, to facilitate air permeability and water impermeability^27^. Figure 3 illustrates the conceptual process of surface modification on aeolian sand to produce hydrophobic sand. By applying a hydrophobic coating to the surface of the hydrophilic raw sand, a hydrophobic sand was obtained^28,29^. The continuous pores generated by the accumulation of loose particles form free airflow channels, which allow air ventilation while the anti-seepage function remains due to the water repellency of sand particles.

## Experiments and methods

### Experimental setup

**Fig. 4:**
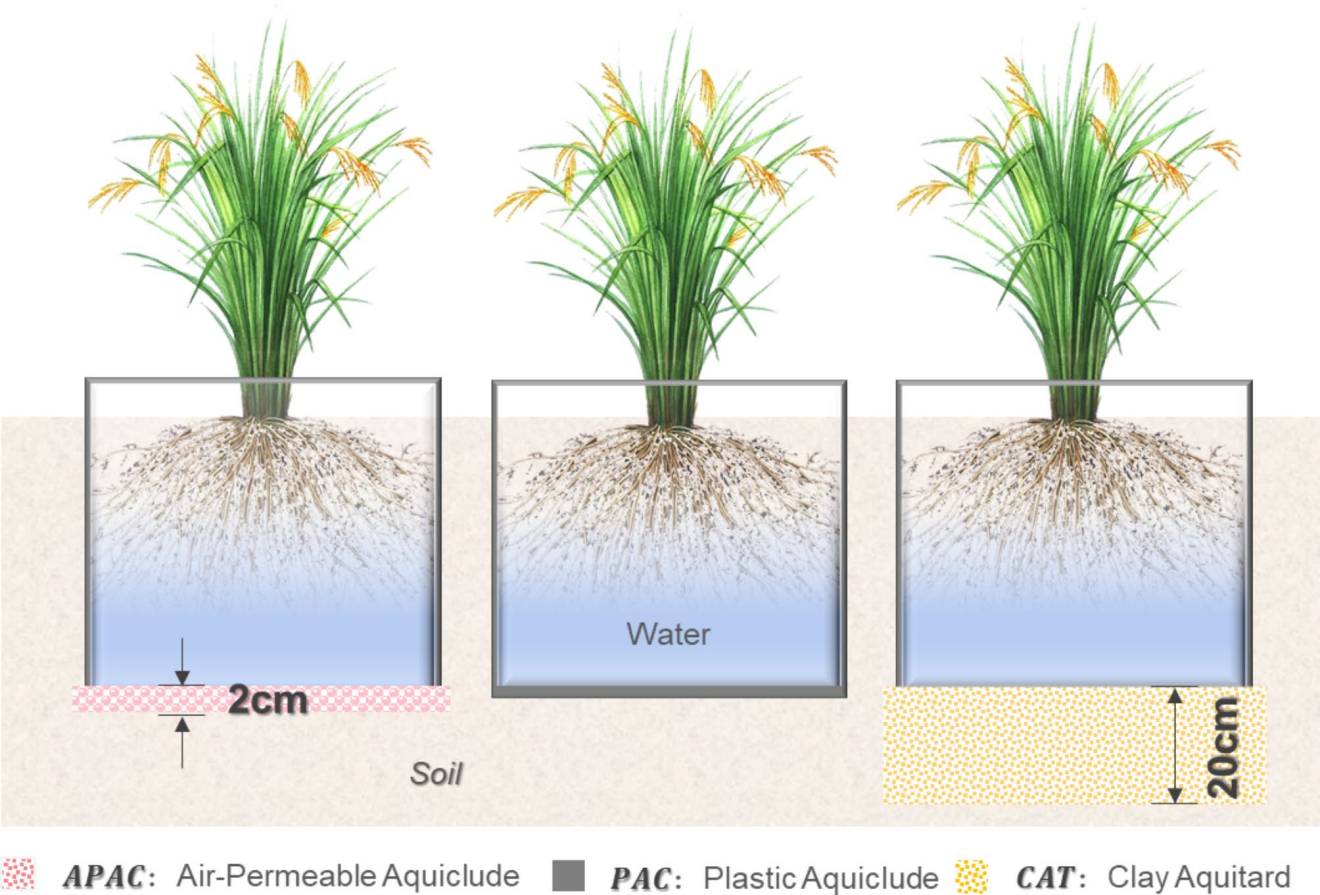
Schematic diagram of the aquiclude structure of the paddy field.

**Fig. 5:**
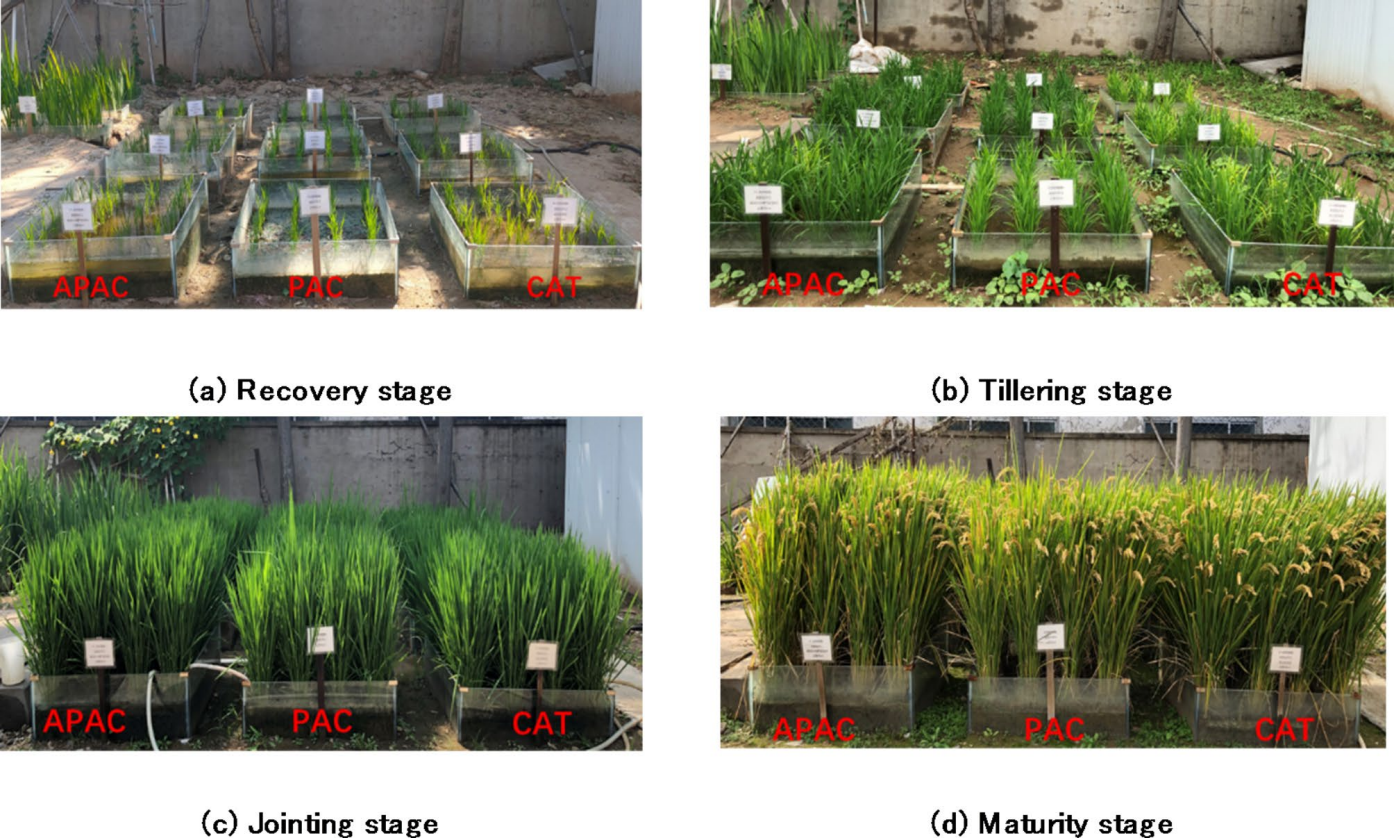
Photograph of the experiment site.

Three seepage reduction types of experiment plots with the same area were tested. All were built under a 45 cm of native soil, as shown in Fig. 4. Air-Permeable Aquiclude (APAC) included a 2 cm thick of hydrophobic sand layer. Plastic Aquiclude (PAC) was built with 2 layers of plastic liner; Clay Aquitard (CAT) was composed of 20 cm thick of compacted clay layer. Three plots were built in each scenario (as shown in Fig. 5). Area was 1 *m*^*2*^ (1 *m* × 1 *m*) in 2021 and (1 *m* × 3 *m*) in 2022, each individual plot was surrounded by impervious berms. Overflow orifices were installed on the berm, 10 cm from the surface of the soil.

### Air permeability and leakage test

Water was added to each plot 0.5 m into each plot after laying three anti-seepage layers. Infiltration volume was measured after 72 h. Using the sandy soil in the test area as a control, the air permeability of the three anti-seepage methods under a pressure difference of 200 p.a. was tested with an air permeability tester (YG461E-II type). The average test results are summarized in Table 1. The data indicates that the air permeability of the APAC is approximately 57% that of sandy soil and about 3.85 times that of the CAT. After the test, 45 *cm* thick sand was backfilled.

**Table 1:** Comparison table of impermeability and air permeability of different aquicludes.

Category	Thickness (cm)	Air permeability (L·m^− 2^·s^− 1^)	Leakage (mm·d^− 1^)
Sandy soil	20	10.65	5760
APAC	2	6.08	0
PAC	-	0	0
CAT	20	1.58	5

### Nitrogen leaching simulation experiment

**Fig. 6:**
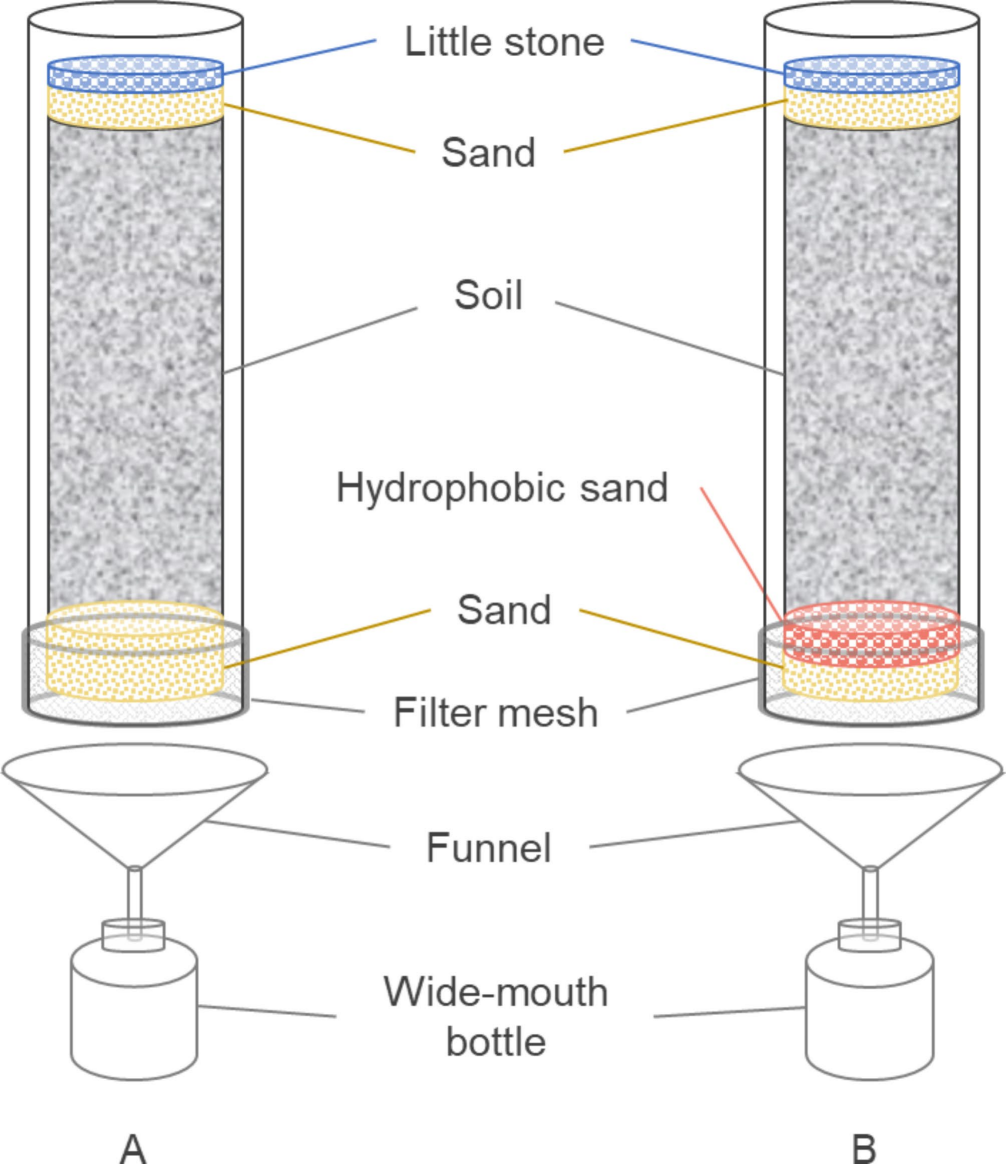
Indoor Simulated Soil Column Leaching Experimental Setup Diagram (A is the blank control group; B is the APAC simulation group).

To assess the nitrogen leaching risk under impermeable APAC conditions, an indoor soil column simulation experiment was conducted (Fig. 6). By measuring the dynamic changes in ammonium nitrogen, nitrate nitrogen, and total nitrogen concentrations in the leachate, we analyzed the characteristics of nitrogen leaching in soil under APAC impermeability conditions.

Surface soil from the experimental planting area was selected as the study subject. Soil was uniformly collected from the surface to a depth of 40 cm, with visible plant roots and stones manually removed. The soil was spread out on a clean, shaded surface to air-dry naturally. After air-drying, the soil was crushed and passed through a 2 mm sieve, ensuring thorough mixing to prevent variability in soil properties from affecting the experimental results. A portion of the soil samples was tested for physicochemical properties before the experiment.

Six transparent acrylic tubes (60 cm in height and 10 cm in diameter) served as containers for soil leaching. The tube bottoms were wrapped in 100-mesh gauze for filtration. Prior to filling, the inner walls of each tube were coated with a thin layer of white Vaseline to increase friction between the soil and tube wall, preventing preferential flow along the tube sides. A 2 cm layer of washed quartz sand (20–40 mesh) was laid at the tube base as a filtering layer to retain soil particles and hydrophobic sand. Three tubes were then filled with 2 cm of hydrophobic sand, while the other three were filled with quartz sand as control samples. To ensure uniform soil porosity, a layered packing method was employed, filling the columns in four increments. After each increment, soil was compacted to approximately 10 cm, and the surface was roughened with a glass rod before adding the next layer, forming a final 40 cm soil column.

Deionized water was applied to each packed soil column, saturating the soil. After two days of sunlit ventilation, leaching began once the moisture content of the 20 cm surface soil reached 20–30%. A layer of gauze was placed on each column to prevent sand separation, and each column’s surface was uniformly treated with 0.8 g of urea based on the soil area. A 2 cm layer of quartz sand (20–40 mesh) and 3 mm washed pebbles were added on top to evenly distribute water and stabilize the column. Funnels with a diameter of 70 mm were affixed to the base of each column, connected to 300 mL wide-mouth bottles for leachate collection.

Deionized water was applied at a rate estimated based on soil moisture content (70–80%), completing irrigation in 10 min. Each leaching cycle concluded when no water droplets fell from the bottom of the soil column. The concentrations of ammonium nitrogen, nitrate nitrogen, and total nitrogen in the collected leachate were measured using the Lian-hua Tech. Co., Ltd. LH-3BN for total nitrogen and the 5B-3B (V8) model for ammonium and nitrate nitrogen. The experiment revealed no nitrogen fertilizer leaching under APAC impermeability conditions.

### Crop management

Each plot was fertilized separately (Table 2). The fertilizers used were controlled-release compound fertilizer (N-P_2_O_5_-K_2_O), urea and potassium dihydrogen phosphate, applied at nitrogen (N) 168*kg·hm*^*-2*^, phosphorus (P) 15 kg*·hm*^*-2*^, and potassium (K) 30 *kg·hm*^*-2*^. Among them, the controlled-release compound fertilizer contains approximately 15.0% nitrogen (N), 6.4% phosphorus (P), and 13.2% potassium (K). Nitrogen fertilization was divided into four stages: basal, tillering, jointing, and panicle, with a mass ratio of 10:4:3:3. Potassium dihydrogen phosphate was applied at a rate of 4 *kg·hm*^*-2*^ at the booting stage.

**Table 2:** Fertilizers management during each growth period of each plot.

Control index		basal	tillering	jointing	panicle	booting stage
2021	Date	Jun.11	Jun.28	Aug.10	Aug.28	Sep.1
	Controlled-release compound fertilizer (g)	22.2	/	/	/	/
	urea(g)	18.1	12	9	9	/
	Potassium dihydrogen phosphate (g)	/	/	/	/	0.4
2022	Date	Jun.16	Jul.12	Aug.22	Sep.2	Sep.5
	Controlled-release compound fertilizer (g)	66.6	/	/	/	/
	urea(g)	54.3	36	27	27	/
	Potassium dihydrogen phosphate (g)	/	/	/	/	1.2

Each plot was managed for drainage and irrigation separately, as outlined in Table 3. Irrigation was stopped one week before harvest, and the water consumption during the rice growth period of each plot was recorded. Pest and weed control measures were strictly implemented throughout the growth period. In 2021, a conventional shallow-water irrigation mode will be adopted, and in 2022, an intermittent irrigation system with alternating shallow and wet conditions was employed.

**Table 3:** Water management during each growth period of rice field.

Control index		*R*-S ^1^	PTT-S ^2^	LT-S ^3^	JB-S ^4^	F-S ^5^	M-S ^6^
2021	Date	Jun.11- Jun.20	Jun.21-Jul.20	Jul.21-Aug.10	Aug.11-Aug.21	Aug.22-Sep.5	Sep.6-Oct.10
	Upper limit of Irrigation (mm)	40	30	D&S-F ^7^	50	30	D-C ^8^
	Lower limit of rain storage (mm)	0	0	D&S-F	0	0	D-C
	Upper limit of rain storage (mm)	50	60	D&S-F ^7^	80	80	D-C
2022	Date	Jun.16-Jun.25	Jun.26-Jul.26	Jul.27-Aug.10	Aug.11-Aug.23	Aug.24-Sep.13	Sep.13-Oct.15
	Upper limit of Irrigation (mm)	40	15	D&S-F	30	15	D-C
	Lower limit of rain storage (mm)	0	0	D&S-F	0	0	D-C
	Upper limit of rain storage (mm)	50	60	D&S-F	80	80	D-C

^1^ R-S: Recovery stage; ^2^ PTT-S: Peak-time of tillering stage; ^3^ LT-S: Latter tillering stage;

^4^ JB-S: Jointing-booting stage; ^5^ F-S: Filling stage; ^6^ M-S: Maturity stage.

^7^ D&S-F: draining and sunning the fields; ^8^ D-C: drought conditions.

### Rice sample collection

#### Collection and processing of plant samples

According to the 5-point sampling method, five holes were selected in each plot at the maturity stage. In each hole, the rice plant was centered on the root, and a soil block of approximately 20 *cm* × 20 *cm* × 20 *cm* was extracted, with the aboveground plant part removed. The selected underground part was placed in a screen bag and soaked in water for 1 h, then rinsed with running water and dried in the shade for 2 h. Excess surface water was absorbed with absorbent paper. Root length and root system volume were measured using a tape measure and the drainage method, respectively. The roots were then de-enzymed at 105 °C for 0.5 *h* and dried at 70 °C to a constant weight. The root dry weight was then measured.

Three main plants were randomly selected from the five holes, and the tiller number and dry weight of the plants were measured. The stems, leaves, and ears of the plants were packaged separately, de-enzymed at 105 °C for 0.5 *h*, dried at 70 °C to constant weight, and then weighed to determine the dry matter.

#### Grain yield of rice and nitrogen content

At the mature stage, the rice from the selected 5-hole was tested for planting. The effective number of panicles, number of grains per panicle, thousand-grain weight, number of filled grains, and seed-setting rate were measured. The remaining rice in the plot was all threshed, dried at 70 °C to constant weight, and then weighed to calculate the actual yield. The rice plant was divided into two parts: straw and grain. Each part was de-enzyme at 105 °C for 0.5 *h* and dried at 70 °C to a constant weight. Twenty grams were selected from each part, crushed, and sieved. The stem Nitrogen content of leaves and grains was then detected using an organic element analyzer, vario EL Cube from Germany.

#### Data processing and statistical analysis

The applied parameters are calculated using the following formulas:

1 $$Plant{\text{ }}nitrogen{\text{ }}accumulation\,=\,total{\text{ }}aboveground{\text{ }}biomass{\text{ }}\left( {dry{\text{ }}weight} \right) \times Nitrogen{\text{ }}concentration$$

2 $$Plant{\text{ }}nitrogen{\text{ }}accumulation\,=\,Straw{\text{ }}nitrogen{\text{ }}accumulation \times Grain{\text{ }}nitrogen{\text{ }}accumulation$$

3 $$Nitrogen{\text{ }}harvest{\text{ }}index,{\text{ }}NHI,{\text{ }}\% {\text{ }}={\text{ }}Grain{\text{ }}nitrogen{\text{ }}accumulation{\text{ }}/{\text{ }}Plant{\text{ }}nitrogen{\text{ }}accumulation \times 100\%$$

4 $$Nitrogengrain{\text{ }}production{\text{ }}efficiency,{\text{ }}NGPE,{\text{ }}kg/kg\,=\,Grain{\text{ }}dry{\text{ }}yield{\text{ }}/Plant{\text{ }}nitrogen{\text{ }}accumulation$$

5 $$Harvest{\text{ }}index,{\text{ }}HI\,=\,Grain{\text{ }}dry{\text{ }}yield{\text{ }}\left( {dry{\text{ }}weight} \right){\text{ }}/{\text{ }}total{\text{ }}aboveground{\text{ }}biomass{\text{ }}\left( {dry{\text{ }}weight} \right)$$

6 $$Nitrogenpartial{\text{ }}factor{\text{ }}productivity,{\text{ }}NPFP\,=\,Grain{\text{ }}dry{\text{ }}yield{\text{ }}/{\text{ }}Applied{\text{ }}fertilizer - nitrogen$$

7 $$Field{\text{ }}water{\text{ }}productivity,{\text{ }}FWP\,=\,{Y_g}/{\text{ }}{Q_z}$$

8 $$Irrigation{\text{ }}water{\text{ }}productivity,{\text{ }}IWP\,=\,{Y_g}/{\text{ }}{Q_g}$$

In (1)-(4), (6), N stands for nitrogen. In (7)-(8), Y_g_ stands for grain dry yield; Q_z_ is the total water consumption of paddy fields from soaking period to harvesting period, in mm; Q_g_ stands for the amount of supplementary water (mm) given to the paddy field by adding water sources, according to a reasonable irrigation method to ensure the normal growth of rice. OriginPro 8.5 was used to process and analyze the experimental data and draw graphs, and SPSS 24.0 was used for significance analysis based on the F-test and the least significant difference (LSD) method. Means were tested by least significant difference at *P* < 0.05 (LSD0. 05).

## Results

### Rice root morphological characteristics

**Fig. 7:**
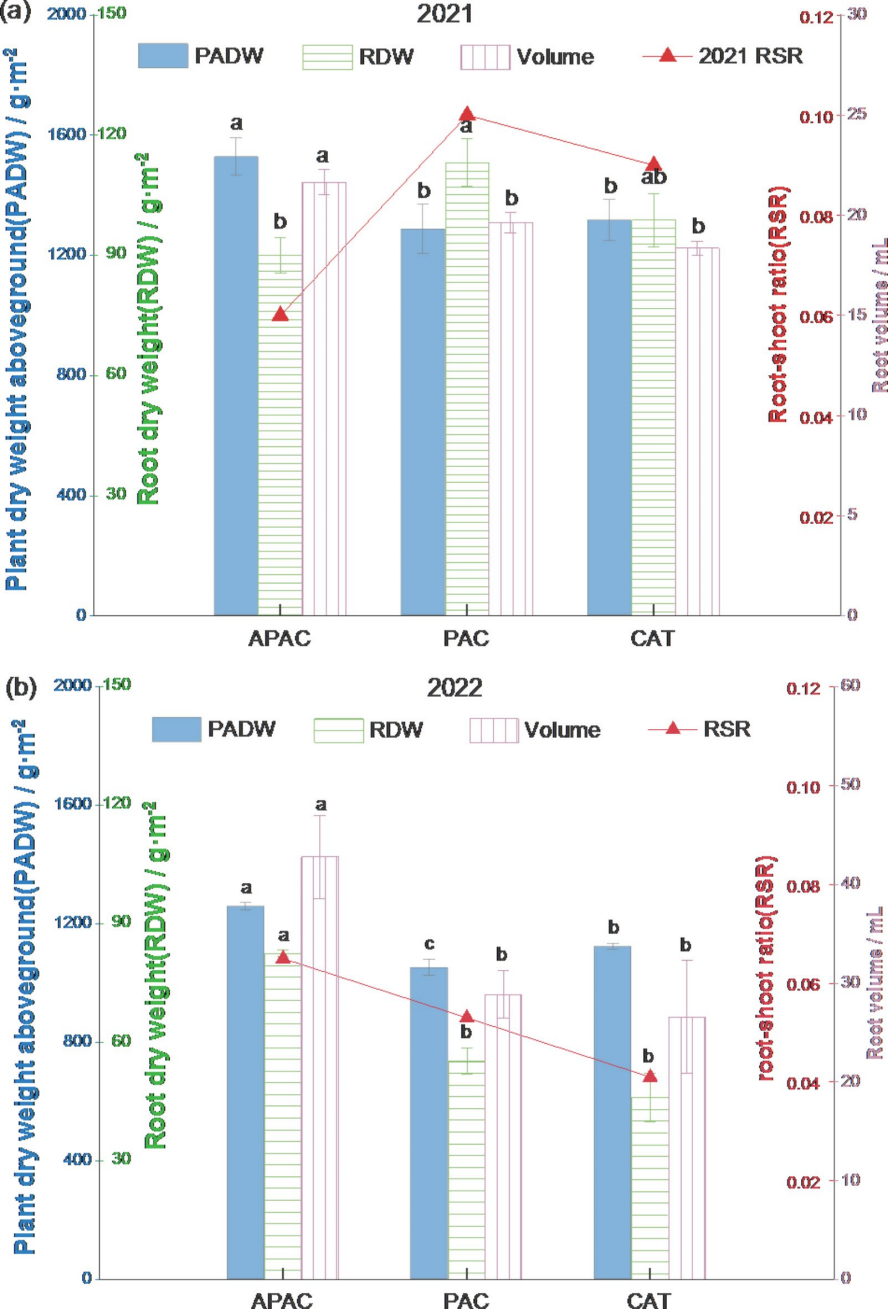
Effects of different models on root morphological characteristics. (Values flanked by different letters in a column mean significant difference at *p* < 0.05 probability level by LSD test.)

As shown in Fig. 7, the APAC exhibits significant in rice root volume, root dry weight, above-ground dry weight, and root-to-shoot ratio, leading to a significant increase in rice root volume and root dry weight. The trend for rice root volume is APAC > PAC > CAT in both years. In 2021, APAC was 10.29% and 17.92% greater than PAC and CAT, respectively. while in 2022, it was 51.77% and 64.79% larger than PAC and CAT, respectively. The above-ground dry weight of rice followed an APAC > CAT > PAC trend in both years. In 2021, APAC increased by 18.77% and 16.03% compared with PAC and CAT, respectively; In 2022, APAC increased by 19.57% and 12.01% compared with PAC and CAT, respectively. The above-ground dry weight of rice followed an APAC > CAT > PAC trend in both years. In 2021, APAC increased by 18.77% and 16.03% compared with PAC and CAT, respectively; In 2022, APAC increased by 19.57% and 12.01% compared with PAC and CAT, respectively. As for the root-to-shoot ratio of rice, in 2021, it exhibited the characteristics of PAC > CAT > APAC, with APAC decreasing by 40.00% and 33.33% compared with PAC and CAT, respectively; In 2022, it showed APAC > PAC > CAT, with APAC increasing by 22.64% and 58.54% compared with PAC and CAT, respectively.

Significant differences can be observed in the morphological characteristics of rice root systems. Compared with 2021, the root volume of APAC increased by 102.31% in 2022, while the root dry weight and aboveground dry weight decreased by 8.35% and 17.60%, respectively. Additionally, the root-to-shoot ratio increased by 8.33%. For PAC, the root system volume increased by 47.02%, but the root dry weight and aboveground dry weight decreased by 51.20% and 19.57%, respectively, leading to a decrease in the root-to-shoot ratio by 47.00%. The root system volume of CAT increased by 44.77%, but the root dry weight and aboveground dry weight decreased by 53.35% and 14.65%, respectively. The crown ratio decreased by 54.44%.

### Rice yields

**Fig. 8:**
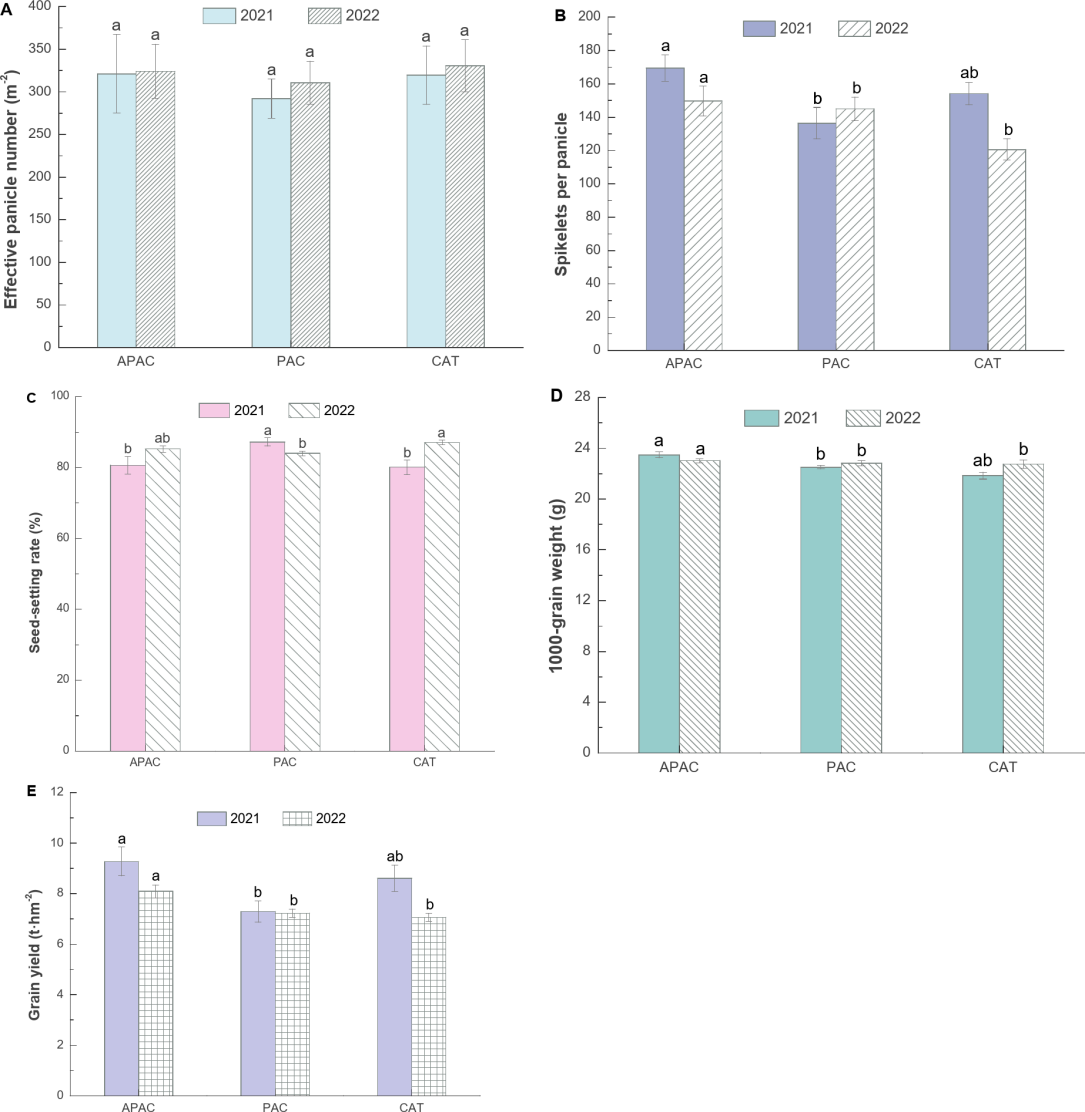
Effects of different treatments on rice yield and its composition. (values flanked by different letters in the same column mean significant difference at *p* < 0.05 probability level by LSD test)

According to Fig. 8, the APAC yield ranges from 8.09 t·hm^-2^ to 9.27 t·hm^-2^, representing an increase of 11.66–27.16% compared with the PAC, and 7.67%~14.52% compared with the CAT. These results indicate that the APAC significantly increases rice yield compared with the PAC. The effective number does not show significant differences among the treatments, with the APAC being 4.28–9.99% higher than the PAC and − 2.00–0.51% higher than the CAT. The number of solid grains per panicle in the APAC increases by approximately 5 to 33 grains compared with the PAC, representing an increase of about 3.19–24.19%. Compared with the CAT, it increases by about 15 to 29 grains, representing an increase of 9.92–24.04%.

The seed setting rate in the APAC is generally slightly lower than in the PAC and CAT, with a decrease of -1.45–7.50% and − 0.77–2.15%, respectively. The thousand-grain weight in the APAC is 0.88–4.36% and 1.23–7.46% higher than in the PAC and CAT, respectively. These results indicate that the APAC’s yield-increasing effect is primarily achieved by increasing the number of solid grains per panicle and the thousand-grain weight.

There were certain differences in yield and yield characteristics over the two years. Compared with 2021, the yields of APAC and CAT were reduced by 8.09% and 13.59%, respectively, in 2022, and the number of solid grains per panicle was reduced by 11.69% and 21.74%, respectively. The number of effective panicles increased by 0.86% and 3.43%, respectively, and the seed setting rate increased by 5.63% and 8.78%, respectively. In contrast, the yield of PAC increased by 4.66%, the number of effective panicles increased by 6.37%, and the number of solid grains per panicle increased by 6.28%, while the seed setting rate decreased by 3.69%.

### Nitrogen Fertilizer partial Productivity(NPFP)

**Fig. 9:**
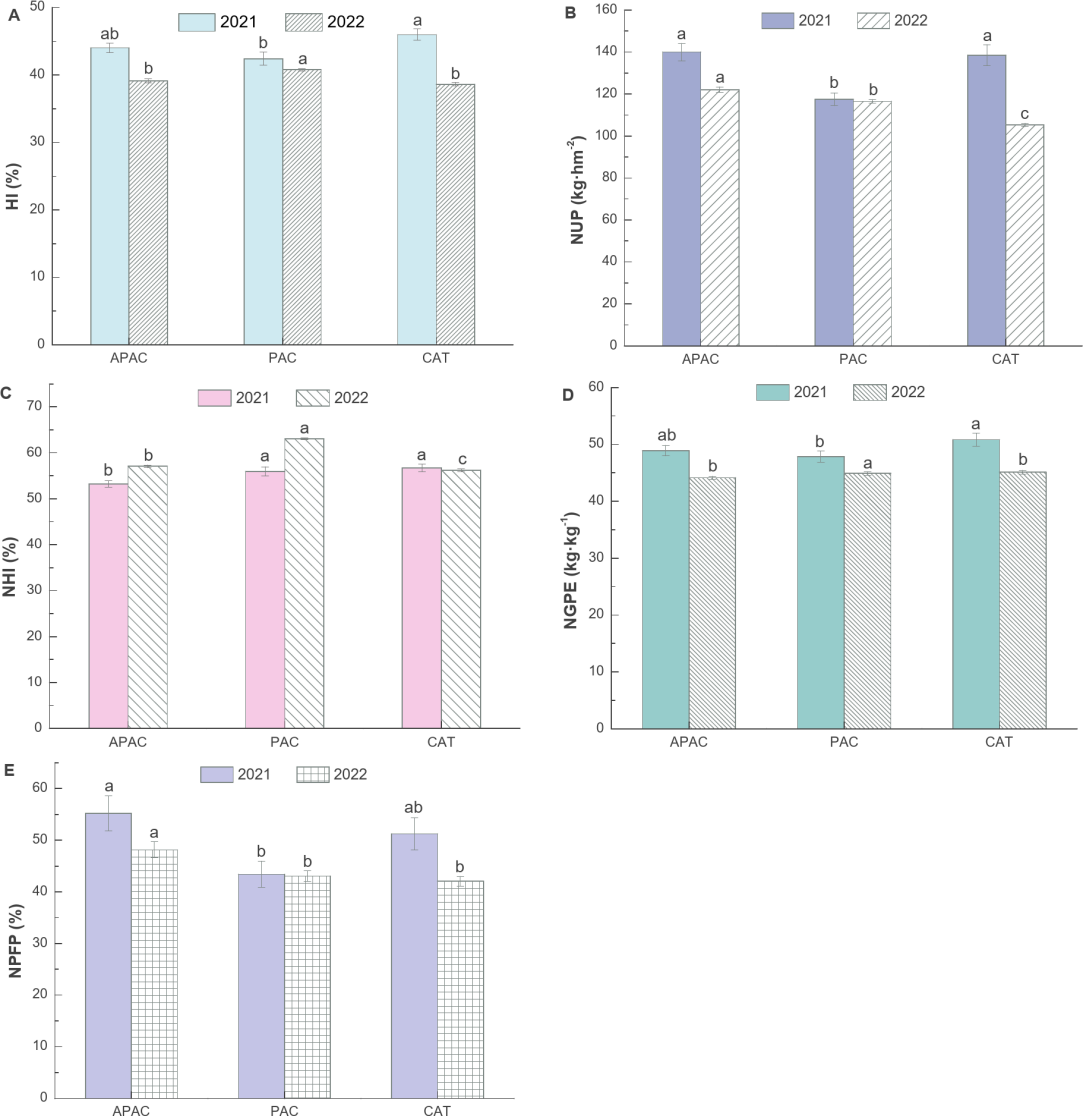
Effects of different treatments on rice yield and its composition. (values flanked by different letters in a column mean significant difference at *p* < 0.05 probability level by LSD test.)

According to Fig. 9, there are significant differences in nitrogen utilization among treatments from 2021 to 2022. The APAC shows significant increases in grain nitrogen accumulation (NUP) and nitrogen fertilizer partial productivity (NPFP). In terms of grain nitrogen accumulation, the APAC increased by 19.18% and 1.05% compared with PAC and CAT, respectively, in 2021, and by 4.81% and 15.98 compared with PAC and CAT in 2022. Regarding nitrogen fertilizer partial productivity, the APAC increased by 19.18% and 1.05% compared with PAC and CAT in 2021 and by 27.06% and 7.69% compared with PAC and CAT in 2022. However, APAC was 1.69% and 2.13% lower than PA and CAT, respectively.

There are significant differences in NHI between APAC, PAC, and CAT. In 2021, the APAC was 4.97% and 6.17% lower than PAC and CAT, respectively. In 2022, APAC was 9.44% lower than PAC and 1.49% higher than CAT. As for the harvest index, the APAC was 3.80% higher than PAC in 2021 and 4.22% lower than CAT. In 2022, the APAC was 40.76% lower than PAC and 1.29% higher than CAT.

Differences in nitrogen utilization over the two years resulted in varying degrees of decrease in grain nitrogen accumulation, nitrogen grain production efficiency, harvest index, and nitrogen partial productivity for each treatment. However, the nitrogen harvest index of APAC and PAC improved. Specifically, for grain nitrogen accumulation, APAC, PAC, and CAT decreased by 12.78%, 0.82%, and 24.00% respectively; for nitrogen partial productivity, APAC, PAC, and CAT decreased by 12.76%, 0.83%, and 17.92% respectively; for nitrogen grain production efficiency APAC, PAC, and CAT decreased by 9.75%, 6.19%, and 11.29% respectively; for harvest index, APAC, PAC, and CAT decreased by 11.11%, 3.87%, and 15.95% respectively; for nitrogen harvest index, APAC and PAC increased by 7.24%, 12.54%, CAT decreased by 0.86%.

### Irrigation water productivity

**Fig. 10:**
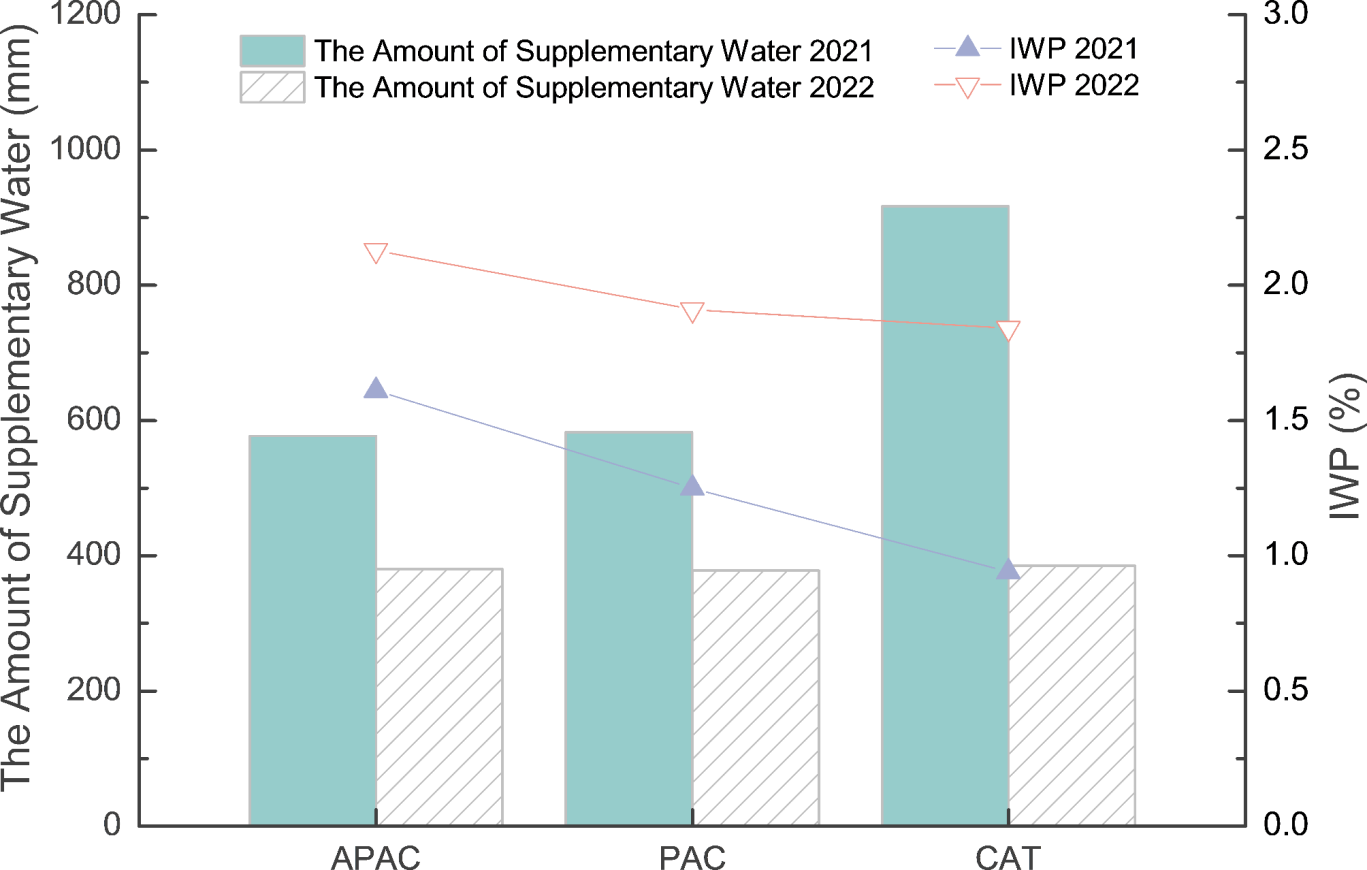
Effects of different models on irrigation water productivity.

Based on Fig. 10, it can be observed that during the flooding irrigation pattern in 2021, APAC and PAC had comparable total irrigation water volumes, while APAC saved 37.08% more water than CAT. In addition, the irrigation water productivity of APAC was higher than PAC and CAT by 28.48% and 71.11%, respectively. Moving on to 2022, under the shallow wet, alternating irrigation pattern, the total irrigation water volumes for all treatments were almost equal. APAC had a higher irrigation water productivity of 11.22% and 16.01% than PAC and CAT, respectively. Furthermore, in 2022, all treatments saved significantly more water than in 2021, with APAC, PAC, and CAT saving 34.14%, 35.21%, and 58.05% more water, respectively. Their irrigation water productivities also increased significantly, with APAC, PAC, and CAT having 1.33, 1.53-, and 1.95-times higher irrigation water productivities, respectively.

## Discussion

The APAC can increase rice yield by 7.67–27.16% (Fig. 8E), perhaps because it has good air permeability and watertightness. Good air permeability can promote air exchange at the soil-atmosphere interface, increase soil oxygen content^18^, and help regulate the balance of water, fertilizer, and air in rice fields. Several studies have shown that moderate soil oxygenation can enhance stress resistance in the middle and late growth stages of rice, promote the rapid development of vegetative organs at the tillering stage, and the rapid formation of tillering and leaf areas^30^, which is beneficial for improving rice growth and rice yield. Additionally, under conventional shallow water irrigation, the yield of the CAT is 18.11% higher than that of the PAC, further illustrating that improving soil permeability is beneficial to increasing rice yield. This study found that the APAC can increase rice yield mainly by increasing the effective number of rice panicles, the number of solid grains per panicle, and the thousand-grain weight. The grain number is 3.19%~24.19%, and the thousand-grain weight is 0.88%~7.46% (Fig. 8). The study found that increasing soil oxygen content can inhibit ineffective rice tillering and improve the degree of rice grain filling^31^. This is also consistent with the results of this study.

The APAC can reduce irrigation water by 37.08% compared with the CAT in 2021 (Fig. 10). It can increase IWP by 11.01–71.11%, closely related to its water-saving and yield-increasing effects. In this research, the amount of transpired water, field drainage, and soil moisture changes under different treatments are similar, with the leakage of the aquicludes determining the difference in field water consumption. Soil moisture control is essential in rice cultivation and management. Using water to regulate fertilizer, air, heat, and drainage is crucial for achieving high yield and efficiency. Research has confirmed that reducing rice field seepage is critical^10,11^. The rice field is an integrated “water-fertilizer-pollution” system. Water saving is closely related to fertilizer saving and emission reduction. Reducing the loss of irrigation water can effectively reduce nitrogen and other fertilizers. Regarding leaching loss, Peng et al.^32^ also confirmed that reducing the leakage of irrigation water in rice fields can effectively reduce the loss of nitrate nitrogen by 45.2 to 73.2%, which is beneficial for improving water production efficiency and reducing sewage discharge.

The APAC has been shown to significantly increase the partial productivity of nitrogen fertilizer by approximately 7.69–27.06% (Fig. 9). Nitrogen absorption and utilization in rice are key factors in yield formation. Nitrogen fertilizer losses in rice fields mainly include leakage, runoff loss, soil ammonia volatilization, and denitrification. There is a significant interaction between soil oxygen content and nitrogen form, which can affect nitrifying microorganisms, denitrifying microorganisms, and anaerobic ammonia-oxidizing microorganisms, thereby influencing the soil nitrogen cycle^33^. Under sufficient nitrogen conditions, increasing soil oxygen content can significantly increase soil ammonification intensity, with root activity also impacting rhizosphere ammonifying capacity^34^. Baram et al.^35^ found that surface and underground aerated drip irrigation can reduce cumulative nitrogen emissions by 37% and 14% by increasing soil oxygen content. Moderate soil oxygenation can promote nitrifying capacity^36^ and improve rice’s absorption and utilization efficiency of nitrogen, phosphorus, and other nutrients^37^. The aforementioned research highlights that the APAC mainly reduces leakage loss and increases soil oxygen content to inhibit nitrogen loss due to denitrification and anaerobic ammonia-oxidizing processes, thereby enhancing the absorption and utilization of nitrogen.

The APAC can increase root volume by 10.29–64.79% (Fig. 7). The soil-microbe-plant root system constitutes a complex ecosystem. The interaction between plants and microorganisms is key to the ecosystem’s impact. Various physical and chemical properties, such as soil temperature, humidity, and oxygen content, interact to affect the rhizosphere microbiome and plant growth status^38,39^. Aquicludes with different air permeability and leakage change soil environmental factors such as soil oxygen and moisture content, affecting the community composition and function of soil and rhizosphere microorganisms, ultimately feeding back to rice growth. This study found that the root volume of the APAC was 10.29–51.77% and 17.92–64.79% larger than that of the PAC and CAT, respectively. Existing studies have found that long-term flooding of paddy fields and poor soil ventilation can easily lead to a long-term low-oxygen environment in the soil, resulting in the accumulation of Fe^2+^, Mn^2+^, etc., leading to rice root rot, poor tiller development, and poor photosynthesis^40^. Increasing soil oxygen content can significantly increase root volume^41^ and the functional period of roots and shoots. It is also noteworthy that the root volume of the PAC is 6.92–8.58% larger than that of the CAT. This is because fertilizer in the CAT seeps and is lost with the water, resulting in insufficient nutrition in the late growth stage of rice, consistent with this research’s conclusion. The dry weight of the root system in the APAC decreased by 8.84–20.39%, and its root-to-shoot ratio decreased by 33.33–40.00%, respectively in 2021, but the dry weight of the plant increased by 16.03–18.77%, indicating that within a certain range, a smaller root crown in the late growth stage of rice is conducive to the reasonable distribution of nutrients, increases dry matter accumulation in the plant and grain, and is conducive to increasing rice yield. Compared with the PAC, the root volume of the CAT decreased by 6.47%, while the aboveground dry weight increased by 2.36%, and the yield increased by 18.11%, which also verifies the above conclusion.

In addition, compared with 2021, the yield of the APAC and CAT decreased by 8.09% and 13.59%, respectively, in 2022 (Fig. 8), and the harvest index, nitrogen fertilizer partial productivity, and nitrogen grain productivity all showed different degrees in the shallow-wet al.ternate irrigation. This indicates that reducing the amount of irrigation water may not be conducive to the absorption and utilization of nitrogen by rice and may easily lead to a reduction in rice yield. Water stress can alter the structure and diversity of microbial communities in the rhizosphere, affecting rice fields’ nitrogen cycle^42^. Further evidence suggests that rice fields under dry planting and aerobic conditions decreased by 27.5% compared to flood irrigation^43^. Bhandari et al.^44^ found that drought stress in rice could reduce physiological traits such as effective tillering, photosynthetic rate, and the number of grains per panicle, decreasing rice yield. Additionally, while drought stress can enhance water use efficiency, it can also significantly reduce yield^45^.

Although this study revealed the significant role of APAC in increasing rice yield, saving water, and improving nitrogen fertilizer use efficiency, there are still some limitations that deserve further exploration. First, the experimental data of this study were based on only two years of field experiments, which failed to fully cover the stability and durability of APAC in long-term applications. For example, the aging or performance degradation of the hydrophobic sand layer may have a long-term effect, especially under extreme climatic conditions such as high temperature and humidity, where the effect of APAC may change. Therefore, long-term follow-up experiments are needed in the future to evaluate the stability and sustainability of APAC under different climatic conditions. Second, although this study explored the effect of APAC on soil oxygen content, the changes in rhizosphere microbial communities and their feedback mechanisms on rice growth are still insufficient. Further research can combine high-throughput sequencing technology to deeply analyze the dynamic changes of APAC on rhizosphere microbial communities at different growth stages, as well as its potential role in rice nutrient absorption, disease prevention, and control, etc. In addition, the effect of APAC on the nitrogen cycle also needs further study to clarify its specific mechanism of action on nitrogen fertilizer use efficiency, especially whether APAC can effectively inhibit nitrogen volatilization and leaching in the long term. Finally, although this study explored the potential of APAC in terms of water conservation and rice yield improvement, it failed to fully consider the environmental benefits of APAC under different irrigation modes, including its impact on greenhouse gas emissions. Therefore, future studies should comprehensively consider the multiple benefits of APAC on rice field ecosystems, such as greenhouse gas emissions and soil organic matter accumulation, to provide a more comprehensive solution for rice field management.

## Conclusion

This study utilizes hydrophobic sand, modified with aeolian sand, as the foundational material for an APAC. The APAC exhibits excellent anti-seepage properties, and an air permeability that can reach 57% of sandy soil, approximately 3.85 times that of the CAT. Compared with the PAC and CAT, the APAC can increase rice yields to 8.09t·hm^-2^ to 9.27 t·hm^-2^, a significant increase of 7.67–27.16%, while saving 37.08% of irrigation water compare with CAT. The APAC mainly increases yield by enhancing the effective number of grains, solid grains per panicle, and thousand-grain weight while reducing irrigation water consumption by minimizing soil leakage. This water-saving and yield-increasing effect enhances irrigation water production efficiency by 11.22–71.11%. Furthermore, the APAC promotes root growth and increases root volume by 10.29–64.79%. In regions with climate conditions suitable for rice cultivation, APAC presents a new technical approach for ecologically reclaiming and enhancing the quality of abandoned lands, such as open-pit abandoned mines. This approach comprehensively achieves the objectives of water and nutrient conservation, emission reduction, and increased yields in rice fields. However, the limitation of this study is that the experimental data is short-lived and there is a lack of systematic evaluation of the long-term application effect and stability of APAC. Future research should further expand the experimental time and spatial scale, combine multi-factor control experiments, and systematically explore the comprehensive impact of APAC on the service functions of rice field ecosystems and their environmental benefits, in order to provide a more comprehensive scientific basis for the innovation of rice planting technology and the efficient ecological restoration of abandoned open-pit mines.

## Electronic supplementary material

Below is the link to the electronic supplementary material.

Supplementary Material 1

Supplementary Material 2

## Data Availability

Data is provided within the manuscript or supplementary information files.

## Abbreviations

APAC: Air-Permeable Aquiclude
PAC: Plastic Aquiclude
CAT: Clay Aquitard
IWP: Irrigation Water Productivity
HI: Harvest index
NHI: Nitrogen harvest index
NUP: grain nitrogen accumulation
NGPE: Nitrogen grain production efficiency
NPFP: Nitrogen Partial Factor Productivity
